# Potentiation of thrombus instability: a contributory mechanism to the effectiveness of antithrombotic medications

**DOI:** 10.1007/s11239-018-1641-2

**Published:** 2018-03-17

**Authors:** Diana A. Gorog

**Affiliations:** 1 National Heart & Lung Institute, Imperial College, Dovehouse Street, London, SW3 6LY UK; 2 Postgraduate Medical School, University of Hertfordshire, Hatfield, UK

**Keywords:** Thrombosis, Platelet aggregation, Endogenous fibrinolysis, Thrombus dispersion, Antithrombotic drugs

## Abstract

The stability of an arterial thrombus, determined by its structure and ability to resist endogenous fibrinolysis, is a major determinant of the extent of infarction that results from coronary or cerebrovascular thrombosis. There is ample evidence from both laboratory and clinical studies to suggest that in addition to inhibiting platelet aggregation, antithrombotic medications have shear-dependent effects, potentiating thrombus fragility and/or enhancing endogenous fibrinolysis. Such shear-dependent effects, potentiating the fragility of the growing thrombus and/or enhancing endogenous thrombolytic activity, likely contribute to the clinical effectiveness of such medications. It is not clear how much these effects relate to the measured inhibition of platelet aggregation in response to specific agonists. These effects are observable only with techniques that subject the growing thrombus to arterial flow and shear conditions. The effects of antithrombotic medications on thrombus stability and ways of assessing this are reviewed herein, and it is proposed that thrombus stability could become a new target for pharmacological intervention.

## Introduction

Platelet aggregation plays a central role in the development of arterial thrombotic events such as myocardial infarction, ischaemic stroke and peripheral arterial thrombosis [1]. Antiplatelet medications such as aspirin and P2Y_12_ inhibitors, which inhibit platelet aggregation, are established as the cornerstone treatment for preventing such thrombotic events [2].

It is generally accepted that antiplatelet agents exert their antithrombotic effects by inhibiting agonist-induced platelet aggregation. It is for this reason that point-of-care platelet function tests that assess platelet aggregation in response to specific agonists, such as arachidonic acid, adenosine diphosphate and thrombin receptor activating peptide have been developed. However, in stenosed arteries, it is a shear gradient-dependent platelet aggregation mechanism which drives thrombus formation, while soluble agonists have only secondary roles, mainly stabilizing the formed aggregate [3].

Further, some important determinants of thrombus formation, such as thrombin generation, fibrinolytic activity and endothelial dysfunction in patients on dual antiplatelet therapy are not determined by platelet aggregability [4]. For example, plasminogen activator inhibitor (PAI-1), a major determinant of fibrinolysis resistance, is released from circulating platelets following platelet activation [5] and yet platelet aggregation is not associated with PAI-1 release [4].

This finding together with the recognition that some patients continue to experience recurrent thrombotic events despite dual antiplatelet medications, has led to the proposal that some patients may benefit from anticoagulant treatment following percutaneous coronary intervention [6].

Amongst patients receiving dual antithrombotic therapy, some patients despite compliance exhibit high on-treatment platelet reactivity (so called “antiplatelet resistance”) and it was shown that this was predictive of recurrent ischaemic events [7, 8]. However, modulation of such high on-treatment platelet reactivity with administration of more potent but still very specific inhibitors of agonist-induced platelet aggregation, failed to translate into a reduction in ischaemic events. Thus on-treatment platelet hyper-reactivity therefore cannot be considered as a risk factor requiring intervention for secondary prevention after percutaneous coronary revascularization [4, 8] and routine platelet function testing to detect high on-treatment platelet reactivity is not advocated [9].

There is however, evidence to suggest that antiplatelet agents may have additional benefits, in addition to inhibition of platelet aggregation, which may impact on thrombus stability.

**Fig. 1:**
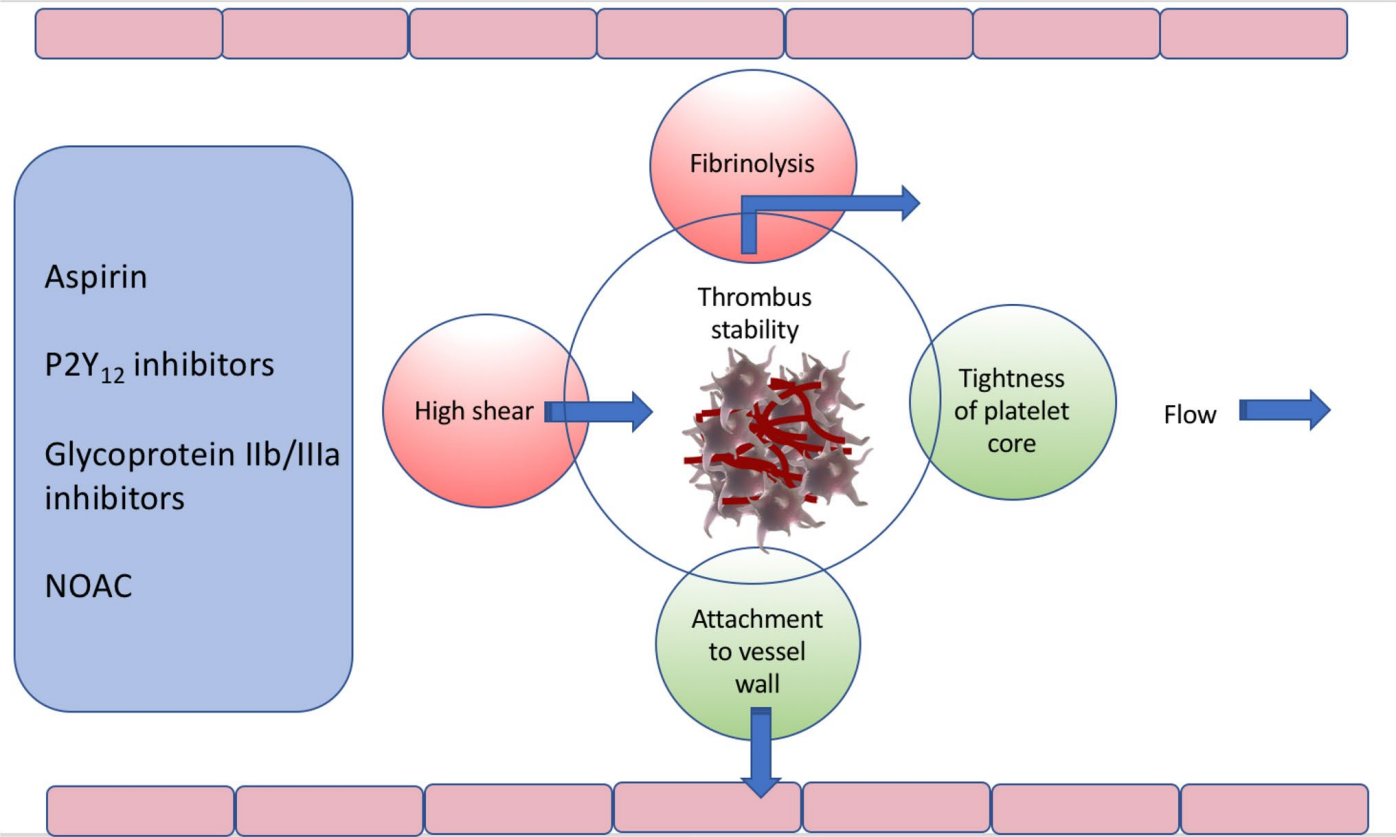
Influences determining arterial thrombus stability, under high shear conditions. The strength of the attachment to the vessel wall and the tightness of the platelet core determine its ability to resist disruption by high shear forces and fibrinolysis. Medications that impact on thrombus stability are shown on left, *NOAC* non vitamin K oral anticoagulant

The stability of an arterial thrombus is the major determinant of the severity and extent of distal tissue damage in myocardial infarction and ischaemic stroke [10]. Thrombus that is unstable is susceptible to fibrinolysis, leading to disruption of the adhesive bonds within the thrombus mass. This, combined with the effects of flow, result in dislodgement or downstream embolization of the thrombus, in whole or in part. On the other hand, a stable thrombus, able to resist fibrinolysis and high arterial back-pressure, will result in potential lasting vessel occlusion with proportionately more extensive tissue damage. The stability of the thrombus, and its ability to withstand dislodgement by high arterial flow, is determined by the combination of the strength of the attachment of the thrombus to the vessel wall, the firmness and the density of the tightly-packed platelet core of the thrombus, and the structure and density of the fibrin network (Fig. 1).

It has been observed that shear-induced platelet aggregation is inversely proportional to shear rate. Shear-induced platelet aggregation occurs when shear-activated platelets aggregate. The intensity of shear rate can regulate both the capture of flowing platelets into the aggregates and the size of those aggregates. Aggregation is less likely to occur at high shear stress sites because hydrodynamic forces carry platelets away with the flow, and aggregation appears to occur predominantly just downstream to this, at location(s) where shear drops but where platelets are able to aggregate via von Willebrand Factor (vWF). Any model involving only the effect of aggregation would inevitably yield to a steady state where all platelets are clumped together in a single aggregate. Hellmuth et al. developed a mathematical kinetic model to better understand the mechanisms of shear-induced platelet aggregation [11]. Previous mathematical models had considered hydrodynamic effects inhibiting platelet aggregation by dislodgement, and incorporated this into models of aggregation efficiency. However, Hellmuth et al. postulated that this alone was too simplistic, since a realistic theoretical steady state can only occur when two opposite forces come to equilibrium. Their model, incorporating not only aggregation, but also disaggregation and breakup processes, implies that aggregates are less noticed at higher shear rates because they break apart very quickly, instead of building up at a slower pace. This mathematical modeling appears to be supported by clinical findings.

Substantial laboratory and clinical data suggest that current antiplatelet and antithrombotic medications exert some of their important effects by potentiating thrombus instability and susceptibility to fibrinolysis (Table 1). The aim of this review is to discuss evidence supporting the assertion that current antithrombotic medications variably, but significantly, reduce thrombus stability and that this in part contributes to their therapeutic effect.

**Table 1:** Effect of various anti-thrombotic drugs on the main determinants of thrombus stability

DRUGS	Fragility of platelet core	Retraction of thrombus	Fibrin structure/fibrinolysis	Non-adherence to endothelium
Aspirin	+		+	+
P2Y_12_ antagonists	+			+
GPI	+	+	+	
NOAC	+		+	
Factor XI inhibitors	+		+	+

*GPI* glycoprotein IIb/IIIa inhibitors, *NOAC* non-vitamin K oral anticoagulant

## Mechanism of thrombus formation at high shear

High shear rate at the arterial wall results in endothelial expression of vWF which results in platelet adhesion via interaction of platelet glycoprotein Ib-IX-V with vWF, with a supporting role for the P-selectin/P-selectin glycoprotein ligand 1 axis. This is followed by firm platelet adhesion to the endothelium via interaction of platelet aIIbb3 with endothelial avb3 and intercellular adhesion molecule 1. Subsequently, thrombin, the thrombospondin-1/CD36 axis and cyclooxygenase 1 all play a role in subsequent platelet activation and thrombus stabilization [12].

**Fig. 2:**
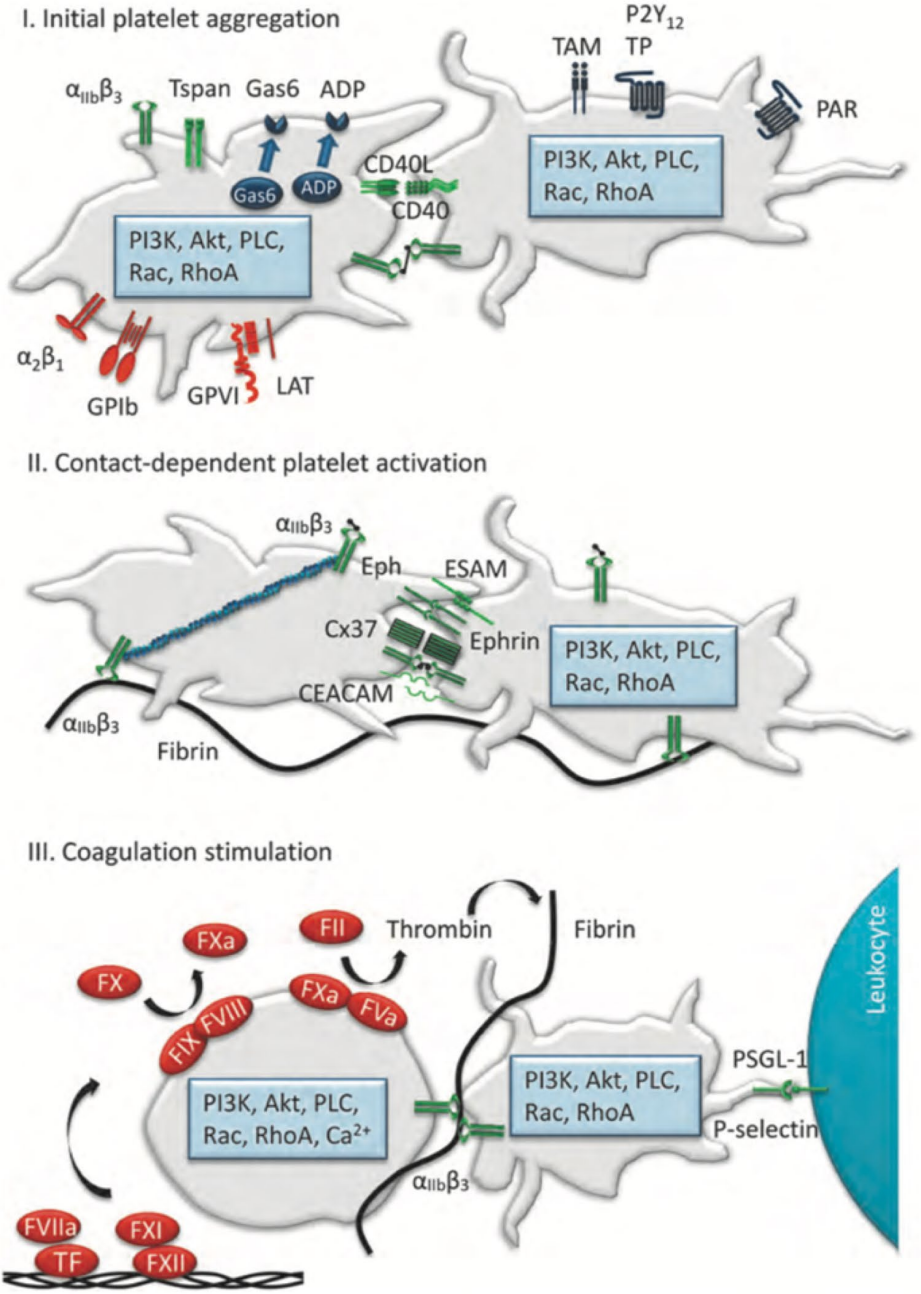
Key platelet and plasma proteins contributing to thrombus stability. **I** Platelet receptors and ligands involved in initial integrina IIbb3 activation and reversible platelet aggregation. The absence of these molecules increases thrombus instability. Also indicated is a box with intracellular signalling proteins controlling this process. **II** Contact-dependent signalling mechanisms implicated in platelet contraction and irreversible platelet aggregation. Fibrin formed by the coagulation process stabilizes the platelet aggregate. **III** Plasma coagulation factors, via the intrinsic (factor XII, FXII) and extrinsic (tissue factor, TF) pathways, mediating platelet-dependent thrombin and fibrin generation, stabilizing a growing thrombus. Also indicated is a primary mechanism of platelet-leucocyte interaction via P-selectin and PSGL-1. Reproduced with permission from Cosemans et al. [14]

Platelet receptors and ligands involved in initial integrin aIIbb3 activation and reversible platelet aggregation are shown in Fig. 2 [13]. As a result of activation, several ligand/receptor pairs and adhesion proteins are expressed on the platelet surface, that form interactions across platelet–platelet contacts thus forming a tightly packed platelet core in the growing thrombus [14].

Contact-dependent signaling mechanisms are implicated in platelet contraction, irreversible platelet aggregation and stabilization of platelet aggregates. Subsequent activation of platelets is further promoted by degranulation and release of adenosine diphosphate (ADP) and thromboxane. Degranulation also results in release of CyPA, a potent stimulant of platelet activation which also enhances binding of fibrinogen to platelets [15]. Thrombin activates platelets and cleaves fibrinogen into fibrin, promoting thrombus growth and stabilization. Fibrin formed by the coagulation process further stabilizes the platelet aggregate. Plasma coagulation factors, via the intrinsic (factor XII, FXII) and extrinsic (tissue factor) pathways, mediating platelet-dependent thrombin and fibrin generation, stabilize the growing thrombus. Thrombin also suppresses its own generation by binding to endothelial-expressed thrombomodulin. Thrombomodulin-bound thrombin activates protein C, which inactivates coagulation factors Va and VIIIa leading to dampening of thrombin generation. The coagulation cascade plays an important role in thrombus formation and stabilization, primarily via thrombin, and secondary feedback loops, among others via thrombospondin-1.

## Impact of antithrombotic medications on thrombus stability

### Aspirin

Although the cardioprotective effects of aspirin are well recognized, after more than three decades of research, the mechanism of aspirin’s antithrombotic effect is still not fully understood. The prevailing view is that aspirin impairs platelet aggregation and subsequent thrombus formation via inhibition of platelet thromboxane synthesis.

However, experimental evidence indicates a significant mismatch between the potent inhibitory effect of aspirin on platelet aggregation measured in vitro in anticoagulated blood, under static conditions, and the magnitude of the effect measured in vivo, under pathologically-relevant flow conditions. This discrepancy suggests that aspirin’s antithrombotic effect may involve other mechanisms of action, in addition to the inhibition of cyclooxygenase.

In a perfusion chamber set-up, aspirin significantly reduced thrombus volume and thrombus height in the presence of citrate but no significant effects were observed in native, non-citrated blood [16–19]. Furthermore, aspirin has been shown to reduce platelet activation only under conditions of low shear stress, with minimal effects at higher shear stress or under dynamic flow conditions [20]. Aspirin’s antithrombotic effect was lost at a pathologically-relevant arterial shear rate of 10,500 s^−1^, and loss of antiplatelet effect at high arterial shear rates has also been demonstrated in patients with severe atherosclerotic stenoses [21]. Perfusion of aspirin-treated blood over endothelium-denuded rabbit aorta in an annular perfusion chamber did not reduce the number of adherent platelets or the subsequent aggregation (thrombus volume) relative to control blood, but the most striking effect of aspirin was the virtual absence of platelet thrombi at the end of perfusion, indicating that aspirin may act by increasing thrombus fragility [22].

It was shown that aspirin directly stimulated the activity of endothelial nitric oxide (NO) synthase and increased bioavailability of systemic NO [23]. The addition of aspirin to blood perfused at high shear rate markedly increased the release of NO from the endothelium into the circulation. This effect was specific to aspirin and not induced by other nonsteroidal anti-inflammatory drugs or by selective cyclo-oxygenase inhibitors. In addition to inhibiting the adherence of platelets to endothelium and subsequent platelet aggregation, NO also inhibits the contractile mechanism in platelets. Contraction of platelets results in the formation of a tightly packed platelet aggregate, which imparts stability to the thrombus and resistance to fibrinolysis [10], and such “clot retraction” makes an important contribution to thrombus stability. By inhibiting the platelet contractile mechanism, NO attenuates “clot retraction”, resulting in reduced stability and micro-embolization of the developing arterial thrombus. In a microfluidic thrombosis model, at high shear rates, aspirin, even at maximum doses of twenty times the recommended daily oral dose, was unable to fully prevent occlusive thrombosis. In contrast, the odds of detachment or embolization were almost five times higher when a sample was treated with aspirin compared to that without aspirin [24].

Using a perfusion chamber to observe the growth and fragmentation of thrombus, it was shown that whilst aspirin or clopidogrel, either alone or in combination, failed to reduce thrombus growth in some individuals; in combination, caused marked destabilization of the formed occlusive thrombus in all subjects [25]. These observations are supported by the finding that aspirin directly alters clot structure, resulting in the formation of clots with thicker fibrin fibres and bigger pores, which are easier to lysis [26, 27].

These laboratory findings, suggesting a mismatch between aspirin’s anti-aggregatory effects and its effects under high shear conditions in native blood, have also been supported by the findings of some recent clinical studies. Although it was postulated that patients with high on-treatment platelet reactivity (HTPR) despite taking aspirin would have a higher risk of adverse cardiovascular events, this postulate has been challenged. A recent study investigating the association between cardiovascular events and the most common laboratory indices of aspirin’s antithrombotic effects, namely HTPR and residual cyclo-oxygenase (COX)-1 activity, found that neither HTPR nor high residual COX-1 activity was significantly associated with likelihood of cardiovascular events [28]. The largest such study so far undertaken, in which platelet aggregation was measured in 900 high-risk patients with stable coronary artery disease taking aspirin as their sole antithrombotic medication, showed that neither HTPR nor thromboxane B_2_ levels were predictive of cardiovascular events over a 3-year follow-up [29].

### P2Y_12_ receptor antagonists

Evidence suggests that there may be two distinct phases in the stabilization of a growing thrombus. The rapid first phase involves the tightening of platelet–platelet contacts in the loose platelet aggregate, resulting in a tight platelet core of thrombus with little space between platelets. Platelet activation during thrombus formation is localized to this central core, surrounded by a shell of non-activated platelets. The second, slower phase is linked to thrombin generation and fibrin formation.

Adenosine diphosphate plays an important role in the formation of a tightly packed, firm platelet core and thus in the maintenance of thrombus stability [30]. The P2Y_12_ receptor is required for sustained activation of integrin αIIbβ3, irreversible platelet aggregation and thrombus stabilization [31]. The important role of continuous signalling by the ADP autocrine loop in the maintenance of thrombus stability, acting through the P2Y_12_ receptor, was confirmed using an ex vivo perfusion chamber technique. Whilst P2Y_12_ receptor antagonist treatment variably reduced the rate of thrombus growth, it consistently reduced thrombus stability and induced thrombus fragmentation [32]. In an in vivo mesenteric artery injury model in P2Y_12_ -null mice, only small unstable thrombi formed which did not reach occlusive size [33].

Electron microscopy revealed that in response to ex vivo ADP stimulation, blood from patients taking clopidogrel formed loosely packed, unstable thrombi, compared to blood from untreated patients, indicating that clopidogrel impaired the formation of the platelet-to-platelet contacts needed for normal thrombus growth and stabilization [34].

In addition to inhibiting peak platelet aggregation in response to ADP, P2Y_12_ antagonists have been shown to greatly enhance subsequent disaggregation through destabilisation of platelet–platelet contacts, and it has been proposed that measurement of disaggregation may be superior to measures of peak aggregation as a way of identifying P2Y_12_ inhibitor non-responsiveness and may allow tailoring of pharmacotherapy [35–37]. In patients on dual antiplatelet therapy comprising aspirin and a P2Y_12_ inhibitor, disaggregation following specific agonist-induced platelet aggregation was variably but significantly increased [38].

Clopidogrel was shown to improve endothelial function and systemic NO bioavailability in patients with coronary artery disease [39]. In contrast to aspirin, this effect appeared to be related to P2Y_12_ receptor inhibition. As described for aspirin, increased synthesis and release of NO from the endothelium affects thrombus stability by weakening the attachment of the thrombus to the vessel wall and by inhibiting clot retraction, leading to enhanced thrombus fragility. Furthermore, thrombus de-stabilisation may be responsible for the benefits of clopidogrel in the setting of therapeutic fibrinolysis. Addition of clopidogrel to aspirin and fibrinolytic therapy in patients with ST-segment elevation myocardial infarction was shown to significantly improve the patency rate of the infarct-related artery and reduced the frequency of ischaemic complications compared to aspirin and fibrinolysis alone [40, 41]. In a model of carotid injury, intravital fluorescence microscopy showed that thrombus formation was markedly unstable in the presence of P2Y_12_ inhibition with ticagrelor or cangrelor. The P2Y_12_-dependent thrombus stabilization was accompanied by increased fibrin binding, and this P2Y_12_ function was restricted to high shear flow conditions [42]. Freshly formed in vitro platelet aggregates were more rapidly dispersed by ticagrelor than control as assessed by light transmission aggregometry and microscopy indicating that signaling via P2Y_12_ may be critical for early platelet thrombus stabilization [43]. An in vivo murine arterial thrombosis model showed that early thrombotic occlusion was partially reversed by intravenous administration of ticagrelor with increased flow and reduction in thrombus size, implying that P2Y_12_ antagonism disrupts the stability of newly formed platelet aggregates, promoting disaggregation [43].

### Glycoprotein IIb/IIIa inhibitors

In addition to the well-recognised effects of glycoprotein IIb/IIIa inhibitors (GPI) in *preventing* platelet aggregation, GPI can also promote instability of pre-formed thrombi by enhancing platelet disaggregation, reducing clot retraction, and reducing soluble CD40 ligand release from platelets [44]. Abciximab, when added after ADP-induced platelet aggregation in vitro, dose-dependently enhanced platelet thrombus disaggregation and could disperse the platelet aggregates down to individual platelets [45], leading to increased fibrin exposure, clot permeability and susceptibility to fibrinolysis [46].

Observation of the three dimensional structure of platelet thrombi formed on a collagen surface, showed that perfusion of blood containing the glycoprotein inhibitors abciximab, eptifibatide, or tirofiban over the formed thrombus resulted in thrombus dissolution, whereas this was not seen with control blood [32, 47]. Such addition of GPI to whole blood under dynamic flow conditions reduced platelet thrombus volume by > 75%, mainly through potentiation of frequent embolization.

Observations from capillary perfusion systems and platelet aggregometry studies showed that abciximab and eptifibatide readily dispersed freshly-formed platelet aggregates, an effect mediated through dissociation of fibrinogen from the platelet surface [48]. Others have suggested that eptifibatide in combination with another antiplatelet agent, but not alone, could enhance thrombus disaggregation [32].

### Non-vitamin K oral anticoagulants (NOACs)

Thrombin, the most potent agonist of platelet aggregation, determines the formation of fibrin and physical characteristics, inhibits fibrinolysis and plays a central role in thrombus growth and stability [49]. Despite the commonality of inhibiting thrombin generation, there would appear to exist important differences between the various non-vitamin K oral anticoagulants (NOACs) in their impact on thrombogenesis and fibrinolysis [50]. The direct thrombin inhibitor dabigatran decreased thrombus stability in a murine model of venous thrombosis through a FXIII-dependent effect [51] and was also shown to enhance the susceptibility of in vitro plasma clots to t-PA-induced lysis, as detected by a turbidometric assay, that was mediated through TAFI activation [52, 53]. This reduction in thrombus stability was not seen with indirect thrombin inhibitors (rivaroxaban and apixaban) when assessed with a turbidometric assay of clot lysis [37]. However, a very recent publication showed that addition of rivaroxaban in vitro enhanced urokinase plasminogen activator activity resulting in enhanced fibrinolytic effects [54]. Urokinase plasminogen activator is a serine protease for plasminogen, the inactive form of plasmin. Activation of plasmin triggers a proteolysis cascade that participates in thrombolysis by increasing fibrinolysis. Using an ex vivo test of thrombosis and thrombolysis, where flowing blood is subjected to high shear, apixaban, rivaroxaban and dabigatran all exhibited a trend toward enhancing endogenous thrombolytic status [55].

## Novel antithrombotic drugs that may impact on thrombus stability

Real-time visualization of thrombus formation in experimental studies has shown that reducing the propagation of the thrombus and reducing its stability may be a novel way of reducing thrombosis. Not surprisingly, a number of novel antithrombotic agents have been developed and hold promise in reducing thrombus stability, discussed in a recent review in detail [56]. These can be broadly grouped into antiplatelet and anticoagulant strategies, and some of those agents that may be particularly effective at altering thrombus stability are discussed below.

### Anticoagulants

Studies using animal models of thrombosis revealed that the contact activation plays an important role in thrombus formation while it has little or no role in haemostasis.

#### Factor XI inhibition

Under pathologically-relevant shear conditions, factor XI has been shown to enhance coagulation, adhesion and aggregation of platelets and thrombus growth on collagen or tissue factor coated surfaces. Factor XI also protected the clot/thrombus against lysis once it was formed [57–59]. In contrast to wild-type mice, mice deficient in factor XI (FXI) failed to form thrombus in a ferric chloride-induced vena cava thrombosis model and exhibited enhanced clot lysis [60]. In animal models of thrombosis, inhibition of FXI enhanced thrombolysis, prevented or reduced thrombus formation and growth, and rendered the thrombus formed more unstable and easily fragmented [32, 61]. In a mouse carotid injury model, treatment with FXI antisense oligonucleotides did not impair initial platelet adhesion and platelet plug formation, but significantly attenuated subsequent thrombus formation and fibrin deposition, with formed thrombi that were much more unstable than thrombi from placebo-treated cohorts [61]. The first FXI-specific strategy to be tested in humans was the subcutaneously-administered FXI-directed antisense oligonucleotide IONIS-416858. In a phase 2 study of 300 patients undergoing elective knee arthroplasty, IONIS-416858 reduced the occurrence of venous thromboembolism and also reduced bleeding compared to enoxaparin [61]. Antisense oligonucleotide, aptamers, antibodies, and small molecules provide a growing armamentarium of agents to reduce thrombosis without increasing bleeding [62].

#### Factor XII inhibition

Inhibition of activated Factor XII (FXIIa) provides thromboprotection by reducing clot firmness [63]. As revealed by intravital microscopy, Thrombi created in injured mesenteric vessels of FXII-deficient mice were unstable and prone to embolization [64]. A recombinant fully human FXIIa activity neutralizing antibody (3F7) has shown promise in animal studies. It has been shown to dose-dependently reduce total and peak thrombin formation [65]. Under flow conditions, 3F7 dose-dependently reduced thrombus formation on collagen and almost completely (< 5% surface covered) abolished thrombus formation at arterial shear rates in a carotid injury model. In an extracorporeal membrane oxygenation cardiopulmonary bypass system in rabbits, 3F7 prevented thrombosis as just as effectively as heparin, but in contrast to heparin, did not did not increase bleeding [65].

### Antiplatelet agents

There are currently no inhibitors in routine clinical use targeting the primary platelet receptors, glycoprotein Ibα or glycoprotein VI, or their ligands (vWF/collagen) that initiate platelet adhesion and activation and become increasingly important as the shear rate increases.

#### Platelet glycoprotein VI inhibition

Inhibition of glycoprotein VI-dependent pathways by interfering in vascular collagen sites could reduce thrombosis in the setting of atherosclerotic plaque rupture, such as in myocardial infarction. Revacept, a soluble dimeric glycoprotein VI-Fc fusion protein has been shown to reduce platelet adhesion by blocking vascular collagen in plaques or erosion and to be safe in preclinical studies. A phase I study in humans showed that Revacept dose-dependently inhibited collagen-induced platelet aggregation without a significant effect on bleeding time. In contrast, ADP- or thrombin receptor activating peptide-dependent platelet aggregation remained unaltered [66].

#### Selective inhibition of platelet inhibition integrin αIIbβ3

Antagonists of the platelet integrin αIIbβ3 are potent anti-thrombotic drugs, but also have the life-threatening adverse effect of bleeding. Integrins transmit signals bidirectionally and outside-in signalling greatly potentiates thrombosis. An inhibitor of Gα13-integrin interaction selectively abolishes outside-in signalling, leading to suppression of thrombosis without affecting bleeding time [31].

#### Inhibition of CyPA

Platelet degranulation results in release of CyPA, a potent stimulant of platelet activation which also enhances binding of fibrinogen to platelets. Inhibition of extracellular CyPA by specific inhibitors, such as MM284 or specific antibody-based antagonists, may modulate thrombus propagation without affecting the haemostasis [15].

## Discussion

Whilst the generally accepted view is that antiplatelet agents exert their antithrombotic effects by inhibiting platelet aggregation, there is ample data from both laboratory and clinical studies to suggest that in addition to inhibiting platelet aggregation, antithrombotic medications in current use increase thrombus fragility and/or enhance endogenous fibrinolysis. Such interference with the mechanisms that normally impart stability to an arterial thrombus and enable it to resist high shear rates, likely contributes to the therapeutic effect of antithrombotic medications. It is not clear how much these effects relate to the measured inhibition of platelet aggregation in response to specific agonists. Most tests of platelet reactivity in clinical use are performed on anticoagulated blood under static conditions or at flow rates that are too low to be pathologically relevant, and therefore cannot assess thrombus stability. This is at least in part why these pharmacological effects of such medications have been relatively overlooked, in comparison to the effects on platelet inhibition, which have been extensively studied. The assessment of the impact of pharmacotherapies on thrombus stability necessitates that the in vitro thrombus be subjected to pathologically-relevant flow and shear conditions. There is now a need for such clinically applicable testing of thrombus stability in individual patients to assess the impact of different antithrombotic medications. Further assessment of these effects may serve to identify new antithrombotic agents which could target thrombus architecture and stability, by affecting platelet packing density and the density of the fibrin meshwork. Already some novel antithrombotic strategies that reduce thrombosis in experimental conditions appear to also reduce thrombus stability. Such targeted pharmacotherapy could reduce lasting thrombotic occlusion.

In conclusion, there is substantial evidence from in vitro and in vivo studies that many antithrombotic medications have shear-dependent effects, potentiating the fragility of the growing thrombus and/or enhancing endogenous thrombolytic activity, and this likely contributes to their clinical effects. The effects of medications on thrombus stability should be further assessed in clinical settings and could become a new pharmacological target.
